# Engineered Endometrial Clear Cell Cancer-on-a-Chip Reveals Early Invasion-Metastasis Cascade of Cancer Cells

**DOI:** 10.34133/bmr.0177

**Published:** 2025-04-14

**Authors:** Chengpan Li, Jing Pan, Zhengdi Shi, Xinyan Zeng, Xiaoping Xia, Xiaogang He, Wei Wang, Bensheng Qiu, Weiping Ding, Dabing Huang

**Affiliations:** ^1^ Department of Electronic Engineering and Information Science, School of Information Science and Technology, University of Science and Technology of China, Hefei, Anhui 230027, China.; ^2^ Department of Oncology, The First Affiliated Hospital of USTC, Division of Life Sciences and Medicine, University of Science and Technology of China, Hefei, Anhui 230001, China.; ^3^ Department of Integrated Traditional Chinese and Western Medicine, Anhui Medical University, Hefei, Anhui 230032, China.; ^4^ Department of Obstetrics and Gynecology, Anhui Provincial Children’s Hospital, Children’s Hospital of Fudan University Anhui Hospital, Children’s Hospital of Anhui Medical University, Hefei, Anhui 230022, China.; ^5^ Department of Urology, The First Affiliated Hospital of USTC, Division of Life Sciences and Medicine, University of Science and Technology of China, Hefei, Anhui 230001, China.

## Abstract

Endometrial clear cell cancer (ECCC) is an extremely rare and highly malignant subtype of endometrial cancer. For most ECCC patients, cancer metastasis is the major cause of death. To date, due to the complexity of cancer evolution and the small number of cases, the metastasis of ECCC at the early stage remains largely unknown. Herein, we modeled the early invasion-metastasis cascade of ECCC by coculturing the ECCC patient-derived tumor cells (PDTCs) and primary human vascular endothelial cells on a microfluidic chip. With the chip, we for the first time replicated the dynamic migration of PDTCs into the surrounding stroma, including the intravasation and extravasation of PDTCs through the capillaries/microvessels, and presented the changes in the morphology and permeability of capillaries, with the decreased diameter and the increased permeability after cancer metastasis. We found that PDTCs were more invasive than the common endometrial adenocarcinoma cells. In addition, we preliminarily explored the inhibition of drugs on the early PDTC infiltration. This study provides new ideas for better understanding of ECCC evolution.

## Introduction

Endometrial cancer is one of the most common gynecologic malignancy in women [1], and endometrial clear cell cancer (ECCC) is an extremely rare estrogen-independent subtype of endometrial cancer [2], accounting for approximately 5% of all endometrial cancer cases [3,4]. Compared with other types of endometrial cancer, ECCC is a high malignancy with rapid growth and high fatality, characterized by the special clear cytoplasm of tumor cells [1,5]. Clinically, most ECCC patients are diagnosed in an advanced stage with myometrial infiltration and distant metastasis [4,6]. Thus, it is of great value to uncover rapid progression mechanism of ECCC for cancer treatment.

Currently, studies on the rare ECCC mainly focus on its histological and pathological characteristics based on the clinical tumor tissue specimens [3,7]. For example, Chang and Ding [8] isolated cancer cells from the ECCC tissues and analyzed the cellular features and gene mutations. However, the dynamic invasion and metastasis of cancer cells in ECCC evolution has still not been dissected [9]. Over past few decades, the widely adopted animal models have greatly advanced cancer research [10]. However, due to the lack of commercial cell lines and sufficient clinical tumor samples, it is difficult to develop tumor-bearing animal models of ECCC. Besides the species differences between animals and humans, it is challenging to visualize the dynamics of cancer cells in ECCC evolution based on animal models [11]. Therefore, it is urgent to develop new strategies to uncover the early invasion and metastasis mechanism of ECCC.

Recently, the emerging organ-on-a-chip provides a novel platform to visually elucidate the complex tumor evolution [12], benefiting from advantages of the controlled tumor microenvironments, observable cellular behaviors, and good biocompatibility [13]. Today, some common cancers have been modeled on chips, such as breast cancer and lung cancer [14]. With the successful construction of on-chip capillaries self-assembled by vascular endothelial cells (ECs) [15,16], the vascularization of on-chip tumors began to be attempted [14], and the interactions between cancer cells and blood vessels were also studied [17,18]. In terms of modeling endometrial disease, microfluidic chips with different patterns have been developed. For example, Gnecco et al. [19] constructed a microfluidic device with a porous membrane to simulate the menstrual cycle under temporal hormone changes. Ahn et al. [20] established a multichannel endometrium chip to study the angiogenesis and hormonal responses of the endometrium. These models greatly facilitate the visualization of the dynamically periodical changes of the endometrium, which advances the clarification of physiological functions of the endometrium; however, on-chip modeling of endometrial cancer, especially the rare ECCC, has rarely been reported [21].

Therefore, in this study, ECCC patient-derived tumor cells (PDTCs) were isolated from the surgically removed tumor tissues, the early invasion-metastasis cascade in ECCC evolution was modeled by coculturing PDTCs and primary human vascular ECs on a microfluidic chip we developed, and the migration of PDTCs and the interactions between tumor cells and capillaries/microvessels were presented. The contributions of this work are as follows: (a) For the first time, we modeled the early-stage invasion-metastasis cascade of ECCC and presented the high aggressiveness of PDTCs on the chip; (b) we revealed the changes in the morphology and permeability of capillaries after the metastasis of PDTCs; (c) we preliminarily tested the outcome of drugs for the inhibition on the infiltration of PDTCs. This work provides a new platform for uncovering the evolution mechanism of ECCC and analyzing potential therapeutics.

## Materials and Methods

### Design and fabrication of the microfluidic chip

In this study, the microfluidic chip we developed is composed of 3 cell culture zones [i.e., upper (U), middle (M), and down (D) channels] and 2 culture medium channels, and 2 adjacent channels are separated by a row of micropillars (Fig. S1). These channels are 100 μm in depth. The chip was fabricated using lithography technology based on the designed patterns [22]. In brief, first, a silicon mold (Suzhou Research Materials Microtech Co. Ltd., Suzhou, China) for the top layer of the chip was manufactured using lithography and etching techniques at the University of Science and Technology of China (USTC) Center for Micro and Nanoscale Research and Fabrication. Then, the silicon mold was placed in a plastic dish, polydimethylsiloxane (PDMS) prepolymer (Dow Corning, Michigan, USA) was poured into the dish to submerge the mold, and the dish was cured in an oven at 85 °C for ~45 min. Next, the patterned PDMS layer was cut out from the dish using a scalpel. Ten through-holes were made at the specified locations of the chip using a microfluidic puncher (Anhui Zhongding Yuxuan New Material Technology Co. Ltd.). Finally, the top PDMS layer was bonded with the bottom cover glass using a plasma bonding machine. To enhance the bonding, the microfluidic chip was placed in an oven at 65 °C overnight. After autoclave sterilization or ultraviolet sterilization, the chips are used for cell experiments. On the chip, the U and D channels were for cell-laden extracellular matrix (ECM), while the M channel was for ECM, and bovine fibrinogen (Yeasen Biotechnology Co. Ltd., Shanghai, China) was used as ECM [14,23].

### Cell culture

In this study, to model the invasion-metastasis cascade in the evolution of ECCC, tumor cells from a young ECCC patient (approved by the Medical Ethics Committee of the Anhui Provincial Children’s Hospital; ethics approval number: EYLL-2022-041), primary human umbilical vein ECs (purchased from Wuhan PriCells Biomedical Technology Co. Ltd.), ECs labeled with red fluorescent protein, and human endometrial adenocarcinoma cell line (HEC-1-A cells; purchased from Beina Chuanglian Biotechnology Co. Ltd.) were used.

**Fig. 1.**
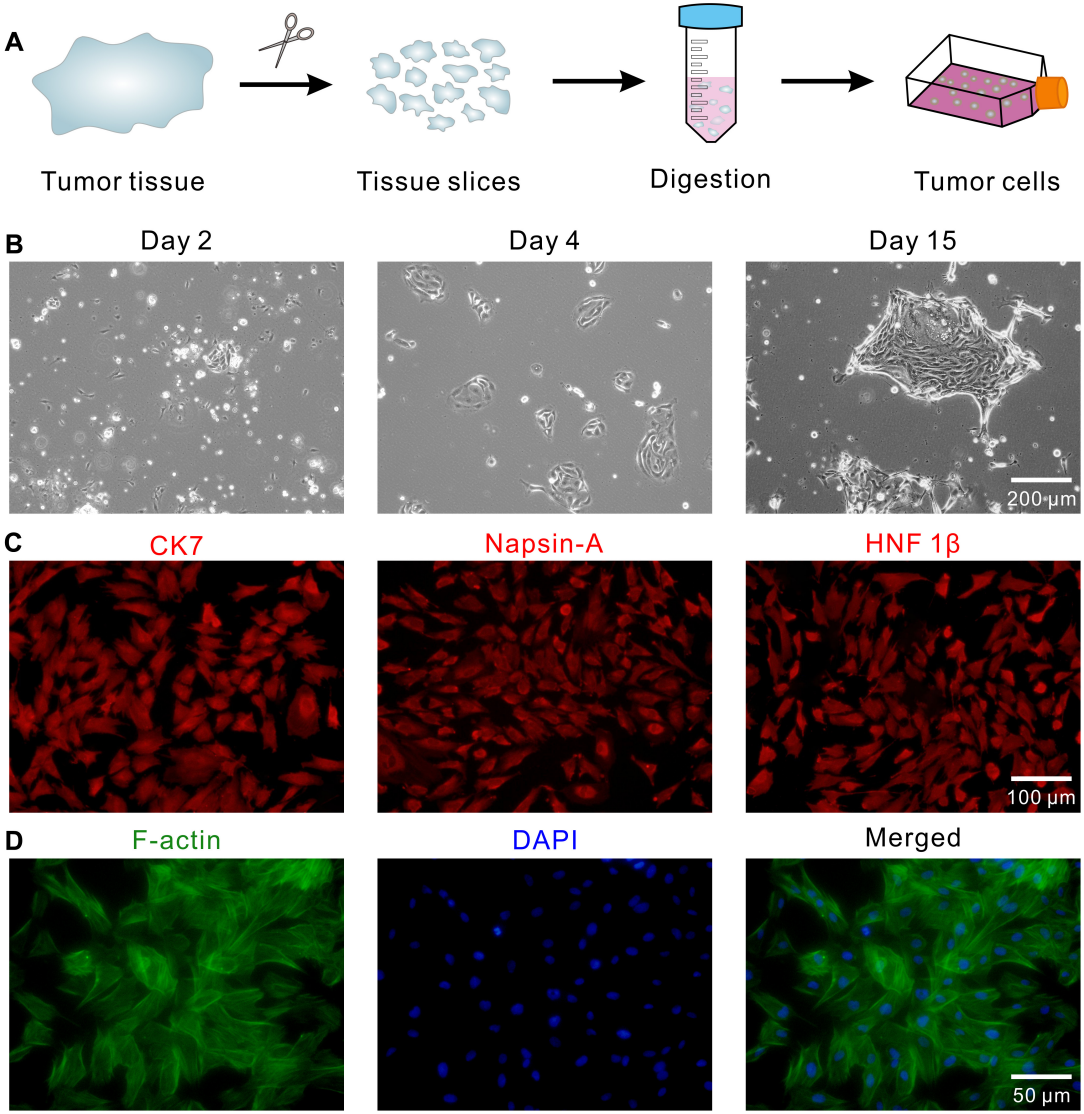
Characterization of PDTCs. (A) Schematic of isolating cells from tumor tissues. (B) Bright-field images of the isolated cells over time. (C) Fluorescence images of PDTCs. Cells were stained with CK7, Napsin-A, and HNF 1β, respectively. (D) Fluorescence images of the cytoskeleton. Cells were stained with phalloidin–FITC (green) and DAPI (blue).

PDTCs were isolated from the surgically removed tumor tissues according to the protocols reported previously (Fig. 1A) [24,25]. In brief, tumor tissues were cut into small pieces and digested using type IV collagenase (1 mg/ml; Beijing Solarbio Science & Technology Co. Ltd., Beijing, China) in the presence of Rock inhibitor (10 μM; SCM075, Beijing Solarbio Science & Technology Co. Ltd., Beijing, China) for approximately 1 h. After centrifugation and filtration, cells were seeded into a cell culture dish. To allow cell attachment, the cells were cultured in Dulbecco’s modified Eagle’s medium (DMEM; Gibco Life Technologies, USA) supplemented with 10% fetal bovine serum (Gibco Life Technologies, USA) and 1% penicillin–streptomycin (Shanghai Beyotime Biotechnology Co. Ltd.) in an incubator at 37 °C and 5% CO_2_ for 24 h, and then DMEM with low glucose supplemented with 1% penicillin–streptomycin was used to culture cells for 48 h (since normal human cells isolated from the tissues will gradually die when cultured in a nutrient-deficient medium within 24 h [26,27]). Thereafter, the cells were kept in DMEM supplemented with 10% fetal bovine serum and 1% penicillin–streptomycin in an incubator at 37 °C and 5% CO_2_. The isolated cancer cells were authenticated by the General Biology (Anhui) Company after comparisons with American Type Culture Collection (ATCC) profiles of short tandem repeats (STRs). To retain the characteristics of patient-derived cells [8], PDTCs passaged 5 to 10 were used for experiments in this study.

ECs were cultured in the EC complete culture medium (ScienCell Research Laboratories, California, USA) in an incubator at 37 °C and 5% CO_2_ [16], and HEC-1-A cells were cultured in DMEM supplemented with 10% fetal bovine serum and 1% penicillin–streptomycin at 37 °C and 5% CO_2_. For the passages of PDTCs, ECs, and HEC-1-A cells, the cells were digested with 0.25% trypsin-EDTA (Gibco Life Technologies, USA) for approximately 3 min at 37 °C, and then the cell suspension was centrifuged for 5 min at 200*g*. After centrifugation, the supernatant was discarded, and cells were cultured in fresh cell culture medium for use.

### Cell loading

First, cells were harvested from the cell culture dishes and suspended in ECM solution (the final concentration of fibrinogen was 2.5 mg/ml; cell density is ~3 × 10^6^ cells/ml). Then, the cell-laden ECM was mixed with thrombin solution (the final concentration was 3 U/ml) and the mixture was rapidly injected into the U/D channel using a pipette (Fig. S1). After loading cells, the chip was placed in an incubator at 37 °C for ~15 min to allow the polymerization of ECM. Next, the mixture of the ECM and thrombin was loaded into the M channel and the chip was placed in an incubator for ~15 min again. Finally, the cell culture medium (50 μl/each) was gently injected into the medium channels to remove air bubbles. It should be noted that to retard polymerization, the operation of loading the mixture of cell-laden ECM/ECM and thrombin was performed on ice. In experiments, the cell culture medium was loaded through medium inlets (IN1: 60 μl; IN2: 40 μl) (Fig. S1), and the flow across the culture zone was driven by the medium column pressure difference [16], with the perfusion flow rate approximately 0.4 μl/h.

### Cell prestaining assay

The dyes DiI and DiO (Shanghai Maokang Biological Technology Co. Ltd., Shanghai, China) were used to stain cells here. After staining with DiI/DiO dye, cells can express red/green fluorescence [28,29]. In brief, cell culture medium was discarded from cell culture dishes, and cells were incubated with medium containing DiI/DiO dyes (3.0 μM) in darkness at 37 °C for approximately 20 min. After incubation, cells were washed with phosphate-buffered saline (PBS) for use. It should be noted that the stained cells need to be kept and operated in darkness in experiments. Here, green fluorescent protein-labeled PDTCs were also used to facilitate the visualization of cell behaviors.

### Cell viability analysis

To confirm cell viability, cells were stained with Hoechst 33342 (Sangon Biotech Co. Ltd., Shanghai, China), calcein-AM (Aladdin Industrial Corporation, Shanghai, China), and propidium iodide (PI; Sangon Biotech Co. Ltd., Shanghai, China) dyes [30]. In brief, cells were incubated with cell culture medium containing Hoechst 33342 (10 μg/ml), calcein-AM (2 μM), and PI (10 μg/ml) at 37 °C for 20 min. After washing with PBS for 3 times, cells were observed under an inverted fluorescence microscope (IX73; Olympus Corporation, Tokyo, Japan), and the fluorescence images were captured. Cell viability was counted using ImageJ software.

### Cytoskeleton staining

To label cytoskeleton, actin-stain 488 conjugated to phalloidin (Shanghai Maokang Biological Technology Co. Ltd., Shanghai, China) was used [31]. In brief, cells were washed with PBS and then fixed with tissue fixative solution (catalog no.: P395744; Aladdin Industrial Corporation, Shanghai, China) for ~40 min. After washing with PBS, cells were permeabilized with 0.2% Triton X-100 (Sangon Biotech Co. Ltd., Shanghai, China) in PBS for ~30 min. The cells were incubated with phalloidin and 4′,6-diamidino-2-phenylindole (DAPI; 10 μg/ml; Shanghai Maokang Biological Technology Co. Ltd., Shanghai, China) dyes for ~30 min in darkness at room temperature. Images were taken using an inverted fluorescence microscope.

### Immunofluorescence staining

For 2-dimensional (2D) immunofluorescence staining, cells were washed with PBS for 3 times and then fixed with tissue fixative solution for ~20 min. After washing with PBST (0.05% Tween 20 in PBS) solution for 3 times, cells were permeabilized with 0.2% Triton X-100 (Sangon Biotech Co. Ltd., Shanghai, China) for ~10 min. Then, cells were treated with 2% bovine serum albumin (BSA; Sangon Biotech Co. Ltd., Shanghai, China) solution for ~2 h in a shaker at room temperature. After washing with PBST solution, cells were incubated with primary antibody [Napsin-A, catalog no.: 60259-2-Ig; hepatocyte nuclear factor 1β (HNF 1β), catalog no.: 12533-1-AP, or cytokeratin 7 (CK7), catalog no.: 17513-1-AP; Proteintech Co. Ltd., Wuhan, China] overnight at 4 °C and then stained with the corresponding CoraLite594-conjugated secondary antibody (catalog no.: SA00013-3/SA00013-4; Proteintech Co. Ltd., Wuhan, China) for ~40 min at room temperature in darkness.

For on-chip immunofluorescence staining [30], medium on the chip was sucked out and the on-chip cells were washed with PBS, fixed with tissue fixative solution for ~40 min, and washed with PBST solution for ~30 min. After treatment with 0.2% Triton X-100 in PBS for ~30 min, the cells were washed with PBST solution and then blocked with 2% BSA solution overnight at 4 °C followed by being washed with PBST solution. Subsequently, the cells were incubated with CD31 antibody (catalog no.: 66065-2-Ig; Proteintech Co. Ltd., Wuhan, China) overnight at 4 °C, washed with PBST solution for ~30 min, and stained with the CoraLite594-conjugated secondary antibody (catalog no.: SA00013-3; Proteintech Co. Ltd., Wuhan, China) for approximately 40 min at room temperature in darkness. After washing with PBST, the fluorescence images of the stained cells were taken using the inverted fluorescence microscope.

### Scanning electron microscope assay

Before experiments, cells were fixed with the tissue fixative for 40 min and washed 3 times with PBS. The fixed cells were dehydrated in order with 10%, 20%, 40%, 60%, 80%, and 100% ethanol for 10 min, respectively [32]. The cells were dried in a critical point dryer (EM CPD300; Leica, Germany), sputtered with gold, and imaged under a scanning electron microscope (EVO18; Zeiss, Germany).

### Vascular permeability assay

Here, fluorescein isothiocyanate (FITC)–dextran solution (70 kDa; 2.5 μg/ml; Xi’an Ruixi Biological Technology Co. Ltd.) was utilized to analyze the perfusion and the diffusive permeability of the on-chip blood vessels [16,33]. Cell culture medium was removed from the chip, and 20 μl of FITC–dextran solution was added to one medium channel. The diffusion permeability of the blood vessels was calculated using ImageJ software based on the fluorescence images according to the equation [32,33]:

$$P_{=}\frac{1}{I_{1}-I_{b}}\left(\frac{I_{2}-I_{1}}{t}\right)\frac{d}{4}$$ (1)

where *P* is the diffusion permeability coefficient, $I_{1}$ is the initial average fluorescence intensity, $I_{2}$ is the final average fluorescence intensity, $I_{b}$ is the background intensity of the images taken before the perfusion of the fluorescent solution, and d is the average diameter of the blood vessel.

### Drug testing assay

The chemotherapeutic drugs (carboplatin and paclitaxel) [5] and the small-molecule inhibitor apatinib targeting the vascular endothelial growth factor receptor family [34] purchased from MedChemExpress Company (Shanghai, China) were used to evaluate the inhibition of drugs on the invasion of PDTCs on the microfluidic chip here. All drugs were dissolved according to operation instructions and added into the cell culture medium. Cells were seeded into the chip and cultured for 48 h at 37 °C and 5% CO_2_, and the on-chip cells were incubated with the cell culture medium containing different drugs for 72 h. During chip culture, images of cells were taken, and data were calculated. Here, the concentrations of carboplatin and paclitaxel were determined based on the drug sensitivity of PDTCs using CCK-8 kit (Shanghai Beyotime Biotechnology Co. Ltd.) according to the manufacturer’s protocol [35]. The concentration of apatinib was determined based on the sensitivity of ECs reported in previous studies [36,37].

### Quantitative polymerase chain reaction analysis

Total RNA was extracted from cells using Trizol reagent from Biosharp Company, cDNA was synthesized using Hifair II 1st Strand cDNA Synthesis Kit (Yeasen Biotechnology Co. Ltd., Shanghai, China), and quantitative reverse transcription polymerase chain reaction (RT-PCR) was performed on the Applied Biosystems Q3 Real-Time PCR System (Applied Biosystems, California, USA) using SYBR Green qPCR Mix kits (Biosharp Company) with the following cycling conditions: 95 °C for 3 min (initial denature) and then 40 cycles of 95 °C for 15 s and 60 °C for 30 s. Glyceraldehyde-3-phosphate dehydrogenase (GAPDH) was used as a housekeeping gene for normalization. The relative change in gene expression was calculated with the 2^−ΔΔCT^ method. The primers used here are as follows: matrix metalloproteinase-9 (MMP9; forward: CCTGGGCAGATTCCAAACCT, reverse: CAAAGGCGTCGTCAATCACC), MKi67 (forward: CAGTTCCACAAATCCAACACA, reverse: GCTGGCTCCTGTTCACGTAT), caspase-3 (Casp-3; forward: TACCTGTGGCTGTGTATCCG, reverse: TTAACGAAAACCAGAGCGCC), E-cadherin (forward: AAGGGGTCTGTCATGGAAGG, reverse: CGTTCAAGGTCAAGACGTGC), and GAPDH (forward: GGAGCGAGATCCCTCCAAAAT, reverse: GGCTGTTGTCATACTTCTCATGG).

### Statistical analysis

In this study, data are presented as the mean ± SD, and *P* values are analyzed using a 2-tailed Student’s *t* test. All the experiments were repeated at least 3 times, and data were from at least 3 samples per test. The significance levels are indicated by * for *P* < 0.05, ** for *P* < 0.01, and *** for *P* < 0.001.

## Results

### Characterization of PDTCs

To obtain tumor cells derived from ECCC patients, cells were isolated from the resected tumor tissues. At initial cell culture stage, there were scattered cells with different morphologies attached to the dish, and the majority of cells were suspended in medium (Fig. 1B). When a low-nutrient medium was used, cells with uniform morphology were clearly captured (only cancer cells can survive in a nutrient-deficient medium [26,27]). After serial cell passages, the hobnail/polygon-shaped cells with the pavement-like arrangement were obtained, which is consistent with previously reported studies on endometrial cancer [8,38]. To further confirm the tumor cells we isolated, the expression of specific proteins (i.e., Napsin-A [39], HNF 1β [40], and CK7 [41]) related to ECCC was confirmed (Fig. 1C), and the cytoskeleton was also presented (Fig. 1D). The integrated analysis showed that cancer cells from the ECCC patient were successfully isolated. In addition, the STR authentication results indicated that PDTCs were not contaminated with any cell lines in ATCC database (Table S1).

### Construction of ECCC-on-a-chip

**Fig. 2.**
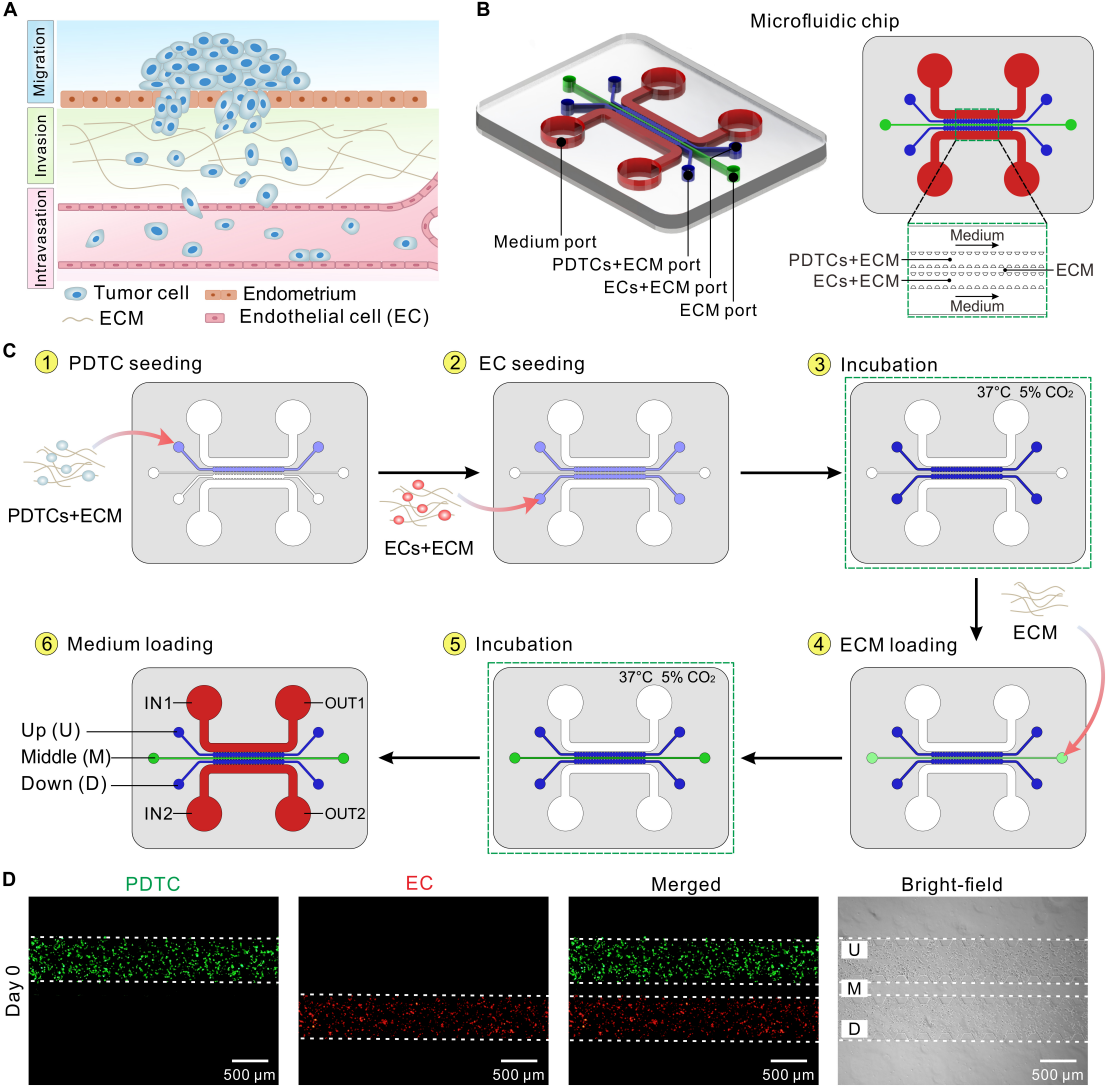
Construction of ECCC-on-a-chip. (A) Schematic of tumor invasion and metastasis. (B) Schematic of the microfluidic chip. (C) Schematic procedure for loading cells into the chip. (D) Images of the loaded cells on the chip. ECs were prestained with DiI (red) dye, and PDTCs were prestained with DiO (green) dye.

To model the early-stage invasion and metastasis of cancer cells in the evolution of ECCC (Fig. 2A), PDTCs and ECs were cocultured on the microfluidic chip (Fig. 2B) after loading PDTC-laden ECM, EC-laden ECM, and ECM into U, D, and M channels, respectively (Fig. 2C). Here, the U channel was for the growth of PDTCs, the M channel was for the migration of PDTCs, and the D channel was for the angiogenesis of ECs. To visualize the location of the loaded cells on the chip, ECs were labeled with red fluorescence, while PDTCs were prestained to express green fluorescence (Fig. 2D). The fluorescence images showed that PDTCs and ECs were successfully seeded into the cell culture zones of the chip without leakage into the lateral channels.

### Viability and characteristics of the on-chip cells

**Fig. 3.**
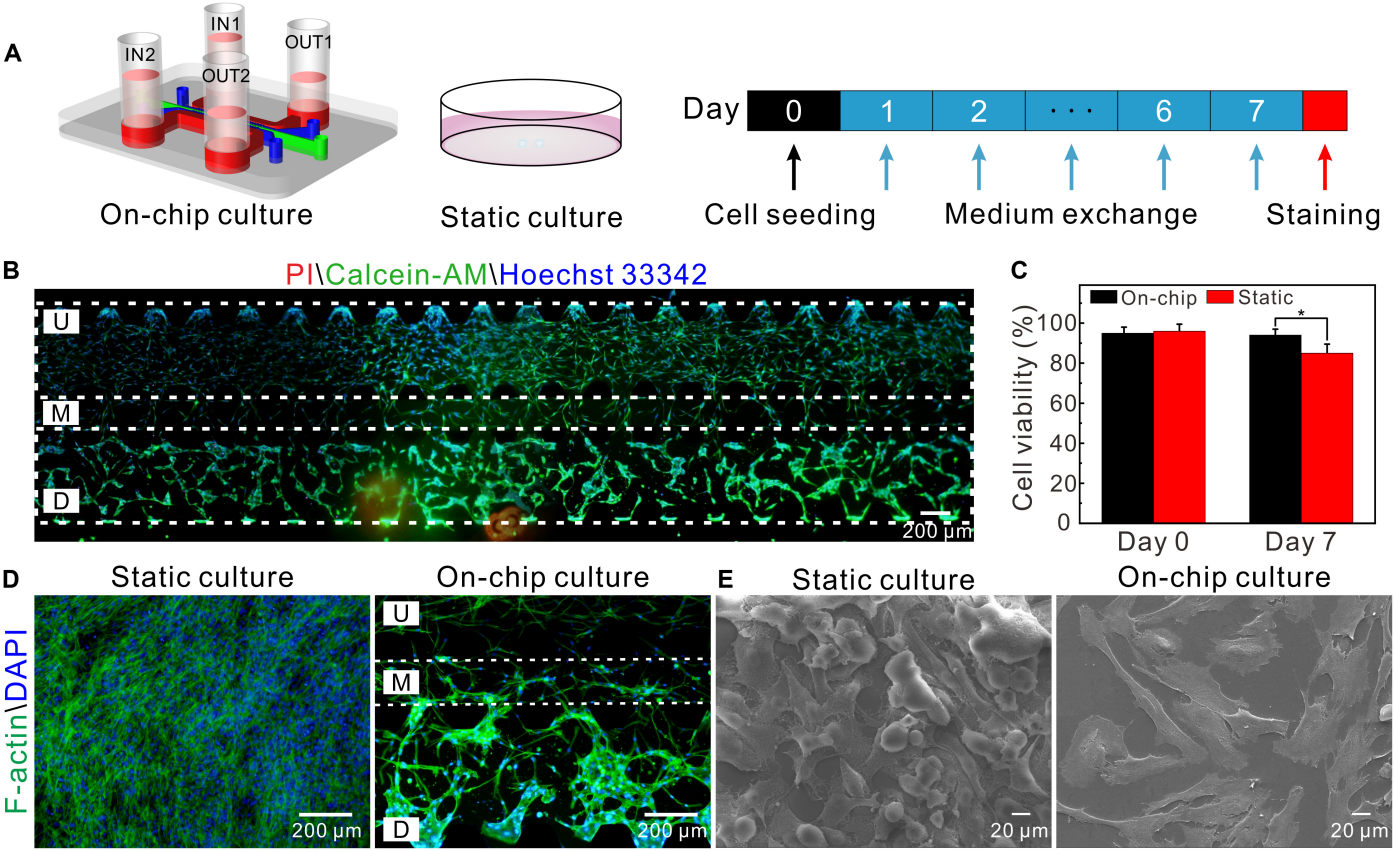
Cell growth under different culture conditions. (A) Schematic of the experiments. (B) Fluorescence images of the cells stained with calcein-AM (green), PI (red), and Hoechst 33342 (blue). (C) Cell viability (*n* = 3). *P < 0.05. (D) Fluorescence images of the cells stained with F-actin (green) and DAPI (blue). (E) Scanning electron microscopy images of PDTCs in static and on-chip groups. For static culture, the cell-laden ECM was seeded on a coverslip in a 48-well plate and the culture medium was added after fibrin polymerization; the volume of the cell-laden ECM (3 μl) and the volume of the medium (200 μl) were the same as the ones in the on-chip culture group, respectively.

To evaluate the growth of the cells on the chip, PDTCs and ECs cocultured on the chip and in cell culture plates (i.e., static culture) were monitored, respectively (Fig. 3A). After cells were cultured for 7 d, cell viability was analyzed (Fig. 3B and Fig. S2). The results showed that cell viability in the static culture group was approximately 80%, whereas in the on-chip group, cell viability was above 90% (Fig. 3C). Compared to the static culture group, the viability of the on-chip cells was higher, benefiting from the flow across the cell culture zone [42]. As the cells gradually proliferated, cells were arranged in a chaotic lattice in the static group (Fig. S3), while on-chip cells stretched and self-assembled into a network, with some cells migrating into the M channel (Fig. 3D). Some on-chip PDTCs presented spindles (Fig. 3E), probably resulting from the transition in morphology of cancer cells for migration [6,43].

### Analysis of the invasion of PDTCs

**Fig. 4.**
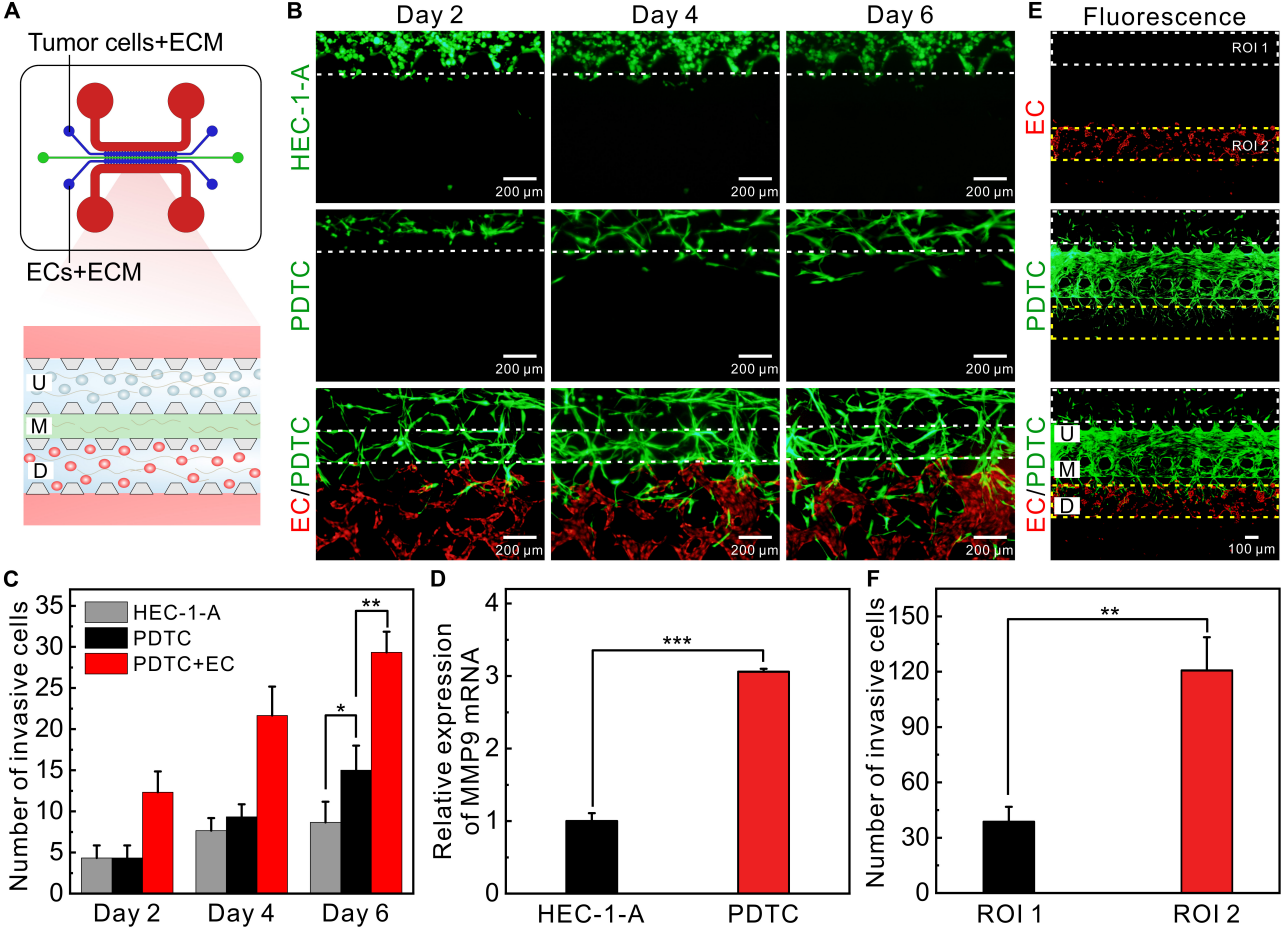
Analysis of the invasion of tumor cells. (A) Schematic of on-chip cells. (B) Fluorescence images of cells on the chip over time. Tumor cells were green, and ECs were red. (C) Number of invasive cancer cells over time (*n* = 3). (D) Relative expression of MMP9 mRNA (*n* = 3). (E) Fluorescence images of on-chip cells on day 7. PDTCs were green, and ECs were red. (F) Number of invasive cells at different regions of interest (ROI; *n* = 4). The data were analyzed using ImageJ. *P < 0.05, **P < 0.01, and ***P < 0.001.

To confirm the invasiveness of endometrial cancer cells, the migration of PDTCs and HEC-1-A cells was analyzed on the chip (Fig. 4A). The results indicated that both PDTCs and HEC-1-A cells gradually invaded into the 3D stroma in the M channel over time (Fig. 4B and Fig. S4); however, the number of the migrated PDTCs is higher than that of the invaded HEC-1-A cells (Fig. 4C). Compared with common endometrial adenocarcinoma cells, the high migration capability of PDTCs may result from the up-regulated mRNA expression of MMP9 (Fig. 4D). Especially, when PDTCs were cocultured with ECs (Fig. S5), both the number of the invasive tumor cells and the migration distance of PDTCs were significantly increased (Fig. 4C and Fig. S6). Interestingly, we found that PDTCs tended to migrate into the side of capillaries/microvessels assembled by ECs, rather than the medium side (Fig. 4E and F). The results suggested that ECs can promote the migration of tumor cells, which is in accordance with previous studies [44,45].

### Analysis of the interactions of PDTCs and capillaries

**Fig. 5.**
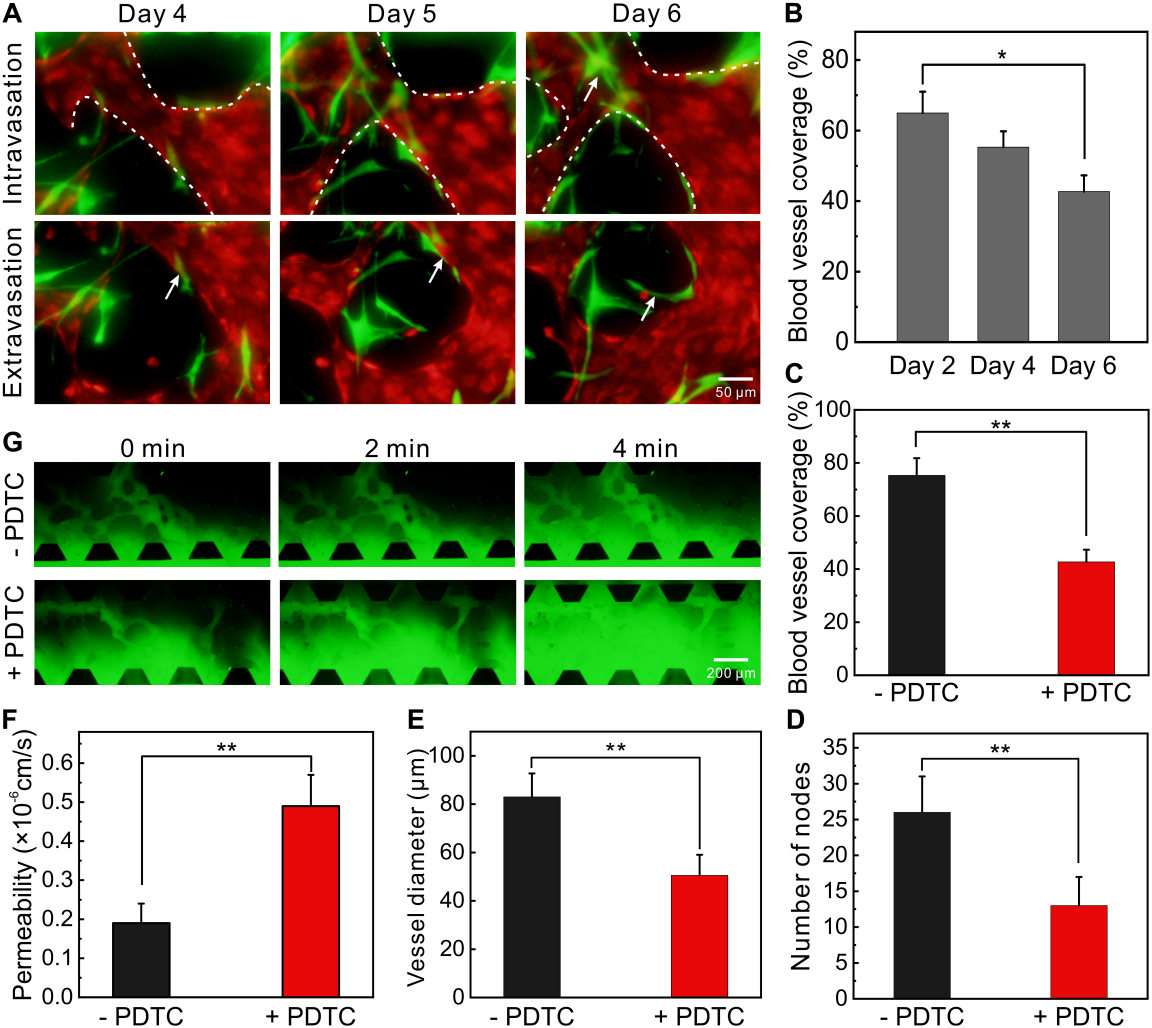
Analysis of the metastasis of PDTCs. (A) Fluorescence images of cells intravasated and extravasated over time (images on days 4 and 6 from locally enlarged subgraphs in Fig. 4B). PDTCs were green, while ECs were red. (B) Coverage of microvessels over time (*n* = 3). (C) Coverage of microvessels in the presence and absence of PDTCs (*n* = 3). (D) Number of the nodes of capillaries in the presence and absence of PDTCs (*n* = 3). (E) Average diameter of microvessels in the presence and absence of PDTCs (*n* = 3). (F) Permeability of microvessels in the presence and absence of PDTCs (*n* = 3). (G) Fluorescence images of capillaries perfused with dextran–FITC solution over time. *P < 0.05 and **P < 0.01.

To analyze the interactions between PDTCs and capillaries, the intravasation and extravasation of PDTCs were presented (Fig. 5A). During on-chip cell culture, the coverage of capillaries decreased with the migration of PDTCs into capillary zone (Fig. 5B). In the presence of PDTCs, the coverage of capillaries was far smaller than the one in the absence of PDTCs (Fig. 5C and Fig. S7), as well as the node number (Fig. 5D) and diameter (Fig. 5E). In addition, the permeability of capillaries increased after PDTC invasion (Fig. 5F and G). Therefore, PDTCs not only changed the morphologies of capillaries but also affected the vascular permeability, and the abnormalities of capillaries in terms of morphologies and the permeability caused by PDTCs indirectly demonstrated the high aggressiveness of ECCC [5].

### On-chip prevention of the invasion of PDTCs

**Fig. 6.**
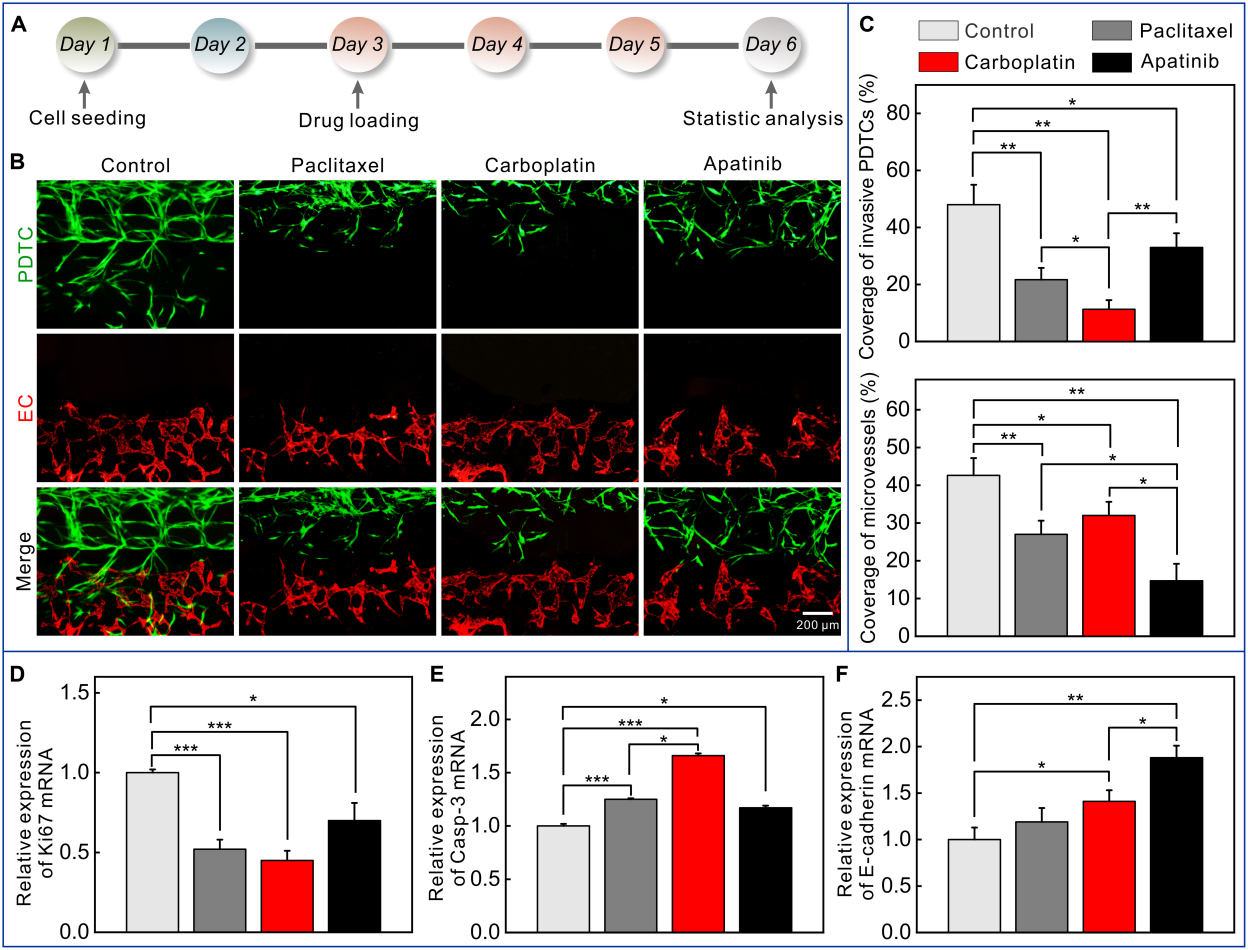
Prevention of the invasion of PDTCs. (A) Protocol for the prevention of the invasion of PDTCs. (B) Fluorescence images of on-chip cells in the control and drug-treated groups. PDTCs were green, and ECs were red. (C) Coverage of the invasive PDTCs and capillaries in the control and drug-treated groups (*n* = 3). (D) Relative expression of Ki67 mRNA (*n* = 3). (E) Relative expression of Casp-3 mRNA (*n* = 3). (F) Relative expression of E-cadherin mRNA (*n* = 3). *P < 0.05, **P < 0.01, and ***P < 0.001.

To prevent the migration/invasion of PDTCs, we conducted the drug experiments on the chip (Fig. 6A). It was observed that both the chemotherapeutic drugs (i.e., paclitaxel and carboplatin) (Fig. S8) and the angiogenesis inhibitor apatinib inhibited the migration of PDTCs (Fig. 6B and Fig. S9). The number of PDTCs that migrated into the lateral stroma was the lowest in the carboplatin-treated group, while the coverage of the capillaries was smallest in the apatinib-treated group (Fig. 6C). Compared to the control, the mRNA expression of Ki67 (a universal biomarker for cell proliferation) was significantly down-regulated (Fig. 6D), and Casp-3 (involvement in cell apoptosis) expression was up-regulated after drug administration (Fig. 6E). However, there was a significant difference in Casp-3 expression between paclitaxel- and carboplatin-treated groups, indicating that cancer cells in this study were more sensitive to platinum-based drugs. In addition, we found that E-cadherin (a migration-associated biomarker) expression was increased after apatinib treatment (Fig. 6F), indicating that apatinib has a great potential for ECCC therapy by inhibiting angiogenesis and cancer cell migration. Collectively, the engineered model is a promising platform for assessing anticancer drugs.

## Discussion

To date, the rapid progression of the rare ECCC has not been uncovered, and the early-stage aggressive behaviors of endometrial cancer clear cells remain unclear [4,6]. In this study, we isolated cancer cells from an ECCC sample and modeled the invasion-metastasis cascade by 3D coculturing the patient-derived cancer cells and vascular ECs on a multichannel microfluidic chip. On the chip, the capillaries self-assembled by ECs were achieved, the dynamic migration of PDTCs into the surrounding stroma and the capillaries was presented, and the changes in morphology and permeability of capillaries after PDTC invasion were analyzed. In addition, the cytotoxicity of the standard first-line chemotherapeutics paclitaxel and carboplatin and the effects of the targeted agent apatinib on cancer cell migration were preliminarily demonstrated. Apatinib suppressed cancer cell migration, which agrees with a phase II clinical trial of apatinib for advanced uterine cancer patients [46]. Thus, the chip platform holds great potentials for evolution mechanism studies and antitumor drug assessment of ECCC. It is important to note that since this study only used cancer cells from one ECCC patient, there may be donor differences in cancer cell behaviors.

Compared to conventional tumor models, microfluidic chips allow real-time analysis of cell–cell interaction and functional quantification assays to check drug responses [47]. Here, due to the limited samples, we aimed to develop an engineered model for studies on the invasion-metastasis cascade of ECCC, and our future work will focus on clarifying the signaling pathways that regulate cancer cell invasion and metastasis and exploiting novel therapeutic targets. For the microfluidic-based model we developed, there is plenty of room for optimization. Here, considering the difficulty of coculture of multiple primary cells, we only loaded vascular ECs and patient-derived cancer cells on the chip. In practice, there are other types of stromal cells in tumor microenvironment, such as tumor-associated fibroblast and immune cells [14,48]. In the future, by optimizing the on-chip coculture methods, more types of stromal cells will be introduced to better replicate the microenvironment of ECCC. In this regard, the combined effects of stromal cells on cancer evolution and treatment can be deeply studied. In addition, to facilitate angiogenesis [14,23], fibrin hydrogel was adopted as ECM on the chip. A highly biomimetic ECM material with multiple protein components needs to be explored in the future.

In conclusion, in this study, we isolated the cancer cells from human ECCC tissues and modeled the early invasion-metastasis cascade in ECCC using the microfluidic chip we fabricated. The high aggressiveness of PDTCs and the changes in capillaries after PDTC invasion were presented. The results showed that PDTCs were more aggressive than endometrial adenocarcinoma cells and that the coverage and permeability of blood vessels were changed after the invasion of PDTCs. In addition, the inhibition of drugs on PDTC invasion were preliminarily evaluated. This study provides new insights into the evolution of endometrial cancer.

## Data Availability

Data will be made available on request.

## References

[1] Crosbie EJ, Kitson SJ, McAlpine JN, Mukhopadhyay A, Powell ME, Singh N. Endometrial cancer. Lancet. 2022;399(10333):1412–1428. 35397864 10.1016/S0140-6736(22)00323-3

[2] Cui P, Cong X, Zhang Y, Zhang H, Liu Z. Endometrial clear cell carcinoma: A population-based study. Front Oncol. 2022;12: Article 961155. 36353550 10.3389/fonc.2022.961155 PMC9638001

[3] DeLair DF, Burke KA, Selenica P, Lim RS, Scott SN, Middha S, Mohanty AS, Cheng DT, Berger MF, Soslow RA, et al. The genetic landscape of endometrial clear cell carcinomas. J Pathol. 2017;243(2):230–241. 28718916 10.1002/path.4947 PMC5708127

[4] Brooks RA, Fleming GF, Lastra RR, Lee NK, Moroney JW, Son CH, Tatebe K, Veneris JL. Current recommendations and recent progress in endometrial cancer. CA Cancer J Clin. 2019;69(4):258–279. 31074865 10.3322/caac.21561

[5] Bogani G, Ray-Coquard I, Concin N, Ngoi NYL, Morice P, Enomoto T, Takehara K, Denys H, Lorusso D, Coleman R, et al. Clear cell carcinoma of the endometrium. Gynecol Oncol. 2022;164(3):658–666. 35063279 10.1016/j.ygyno.2022.01.012

[6] Chaffer CL, Weinberg RA. A perspective on cancer cell metastasis. Science. 2011;331(6024):1559–1564. 21436443 10.1126/science.1203543

[7] Hoang LN, McConechy MK, Meng B, McIntyre JB, Ewanowich C, Gilks CB, Huntsman DG, Köbel M, Lee C-H. Targeted mutation analysis of endometrial clear cell carcinoma. Histopathology. 2015;66(5):664–674. 25308272 10.1111/his.12581

[8] Chang Y-H, Ding D-C. A clear cancer cell line (150057) derived from human endometrial carcinoma harbors two novel mutations. BMC Cancer. 2020;20(1):1058. 33143664 10.1186/s12885-020-07567-w PMC7607743

[9] Weiss F, Lauffenburger D, Friedl P. Towards targeting of shared mechanisms of cancer metastasis and therapy resistance. Nat Rev Cancer. 2022;22(3):157–173. 35013601 10.1038/s41568-021-00427-0 PMC10399972

[10] Mendes N, Dias Carvalho P, Martins F, Mendonça S, Malheiro AR, Ribeiro A, Carvalho J, Velho S. Animal models to study cancer and its microenvironment. In: Serpa J, editor Tumor microenvironment: The main driver of metabolic adaptation. Cham: Springer International Publishing; 2020. p. 389–401. 10.1007/978-3-030-34025-4_20 32130710

[11] Robinson NB, Krieger K, Khan FM, Huffman W, Chang M, Naik A, Yongle R, Hameed I, Krieger K, Girardi LN, et al. The current state of animal models in research: A review. Int J Surg. 2019;72:9–13. 31627013 10.1016/j.ijsu.2019.10.015

[12] Rodrigues J, Heinrich MA, Teixeira LM, Prakash J. 3D in vitro model (R)evolution: Unveiling tumor–stroma interactions. Trends Cancer. 2021;7:249–264. 33218948 10.1016/j.trecan.2020.10.009

[13] Yan J, Li Z, Guo J, Liu S, Guo J. Organ-on-a-chip: A new tool for in vitro research. Biosens Bioelectron. 2022;216: Article 114626. 35969963 10.1016/j.bios.2022.114626

[14] Li C, Holman JB, Shi Z, Qiu B, Ding W. On-chip modeling of tumor evolution: Advances, challenges and opportunities. Mater Today Bio. 2023;21: Article 100724. 10.1016/j.mtbio.2023.100724 PMC10359640 37483380

[15] Kim S, Lee H, Chunga M, Jeon NL. Engineering of functional, perfusable 3D microvascular networks on a chip. Lab Chip. 2013;13:1489–1500. 23440068 10.1039/c3lc41320a

[16] Wang X, Phan DTT, Sobrino A, George SC, Hughes CCW, Lee AP. Engineering anastomosis between living capillary networks and endothelial cell-lined microfluidic channels. Lab Chip. 2016;16(2):282–290. 26616908 10.1039/c5lc01050k PMC4869859

[17] Wong BS, Shah SR, Yankaskas CL, Bajpai VK, Pei-Hsun W, Chin D, Ifemembi B, ReFaey K, Schiapparelli P, Zheng X, et al. A microfluidic cell-migration assay for the prediction of progression-free survival and recurrence time of patients with glioblastoma. Nat Biomed Eng. 2021;5(1):26–40. 32989283 10.1038/s41551-020-00621-9 PMC7855796

[18] Nam H, Funamoto K, Jeon JS. Cancer cell migration and cancer drug screening in oxygen tension gradient chip. Biomicrofluidics. 2020;14: Article 044107. 32742536 10.1063/5.0011216 PMC7375834

[19] Gnecco JS, Pensabene V, Li DJ, Ding T, Hui EE, Bruner-Tran KL, Osteen KG. Compartmentalized culture of perivascular stroma and endothelial cells in a microfluidic model of the human endometrium. Ann Biomed Eng. 2017;45(7):1758–1769. 28108942 10.1007/s10439-017-1797-5 PMC5489603

[20] Ahn J, Yoon MJ, Hong SH, Cha H, Lee D, Koo HS, Ko JE, Lee J, Oh S, Jeon NL, et al. Three-dimensional microengineered vascularised endometrium-on-a-chip. Hum Reprod. 2021;36(10):2720–2731. 34363466 10.1093/humrep/deab186 PMC8450871

[21] Murphy AR, Campo H, Julie Kim J. Strategies for modelling endometrial diseases. Nat Rev Endocrinol. 2022;18(12):727–743. 36050476 10.1038/s41574-022-00725-z PMC10052865

[22] Faustino V, Catarino SO, Lima R, Minas G. Biomedical microfluidic devices by using low-cost fabrication techniques: A review. J Biomech. 2016;49(11):2280–2292. 26671220 10.1016/j.jbiomech.2015.11.031

[23] Phang SJ, Basak S, Teh HX, Packirisamy G, Fauzi MB, Kuppusamy UR, Neo YP, Looi ML. Advancements in extracellular matrix-based biomaterials and biofabrication of 3D organotypic skin models. ACS Biomater Sci Eng. 2022;8(8):3220–3241. 35861577 10.1021/acsbiomaterials.2c00342

[24] Kito F, Oyama R, Noguchi R, Hattori E, Sakumoto M, Endo M, Kobayashi E, Yoshida A, Kawai A, Kondo T. Establishment and characterization of novel patient-derived extraskeletal osteosarcoma cell line NCC-ESOS1-C1. Hum Cell. 2020;33(1):283–290. 31625124 10.1007/s13577-019-00291-z

[25] Berg HF, Hjelmeland ME, Lien H, Espedal H, Fonnes T, Srivastava A, Stokowy T, Strand E, Bozickovic O, Stefansson IM, et al. Patient-derived organoids reflect the genetic profile of endometrial tumors and predict patient prognosis. Commun Med. 2021;1:20. 35602206 10.1038/s43856-021-00019-x PMC9053236

[26] Izuishi K, Kato K, Ogura T, Kinoshita T, Esumi H. Remarkable tolerance of tumor cells to nutrient deprivation: Possible new biochemical target for cancer therapy. Cancer Res. 2000;60(21):6201–6207. 11085546

[27] Sato K, Tsuchihara K, Fujii S, Sugiyama M, Goya T, Atomi Y, Ueno T, Ochiai A, Esumi H. Autophagy is activated in colorectal cancer cells and contributes to the tolerance to nutrient deprivation. Cancer Res. 2007;67(20):9677–9684. 17942897 10.1158/0008-5472.CAN-07-1462

[28] Li Y, Song Y, Zhao L, Gaidosh G, Laties AM, Wen R. Direct labeling and visualization of blood vessels with lipophilic carbocyanine dye DiI. Nat Protoc. 2008;3(11):1703–1708. 18846097 10.1038/nprot.2008.172 PMC2811090

[29] Honig M, Hume RI. Dil and DiO: Versatile fluorescent dyes for neuronal labelling and pathway tracing. Trends Neurosci. 1989;12:333–341. 2480673

[30] Du X, Zou R, Du K, Huang D, Miao C, Qiu B, Ding W, Li D. Modeling colorectal cancer-induced liver portal vein microthrombus on a hepatic lobule chip. ACS Appl Mater Interfaces. 2023;15:56859–56868. 10.1021/acsami.3c14417 38033197

[31] Sticker D, Rothbauer M, Lechner S, Hehenberger MT, Ertl P. Multi-layered, membrane-integrated microfluidics based on replica molding of a thiol-ene epoxy thermoset for organ-on-a-chip applications. Lab Chip. 2015;15(24):4542–4554. 26524977 10.1039/c5lc01028d

[32] Dai Z, Li C, Shi Z, Li S, Luo T, Ding W. A glomerulus chip with spherically twisted cell-laden hollow fibers as glomerular capillary tufts. Biofabrication. 2023;15(3): Article 035004. 10.1088/1758-5090/acc35d 36898152

[33] Nagaraju S, Truong D, Mouneimne G, Nikkhah M. Microfluidic tumor-vascular model to study breast cancer cell invasion and intravasation. Adv Healthc Mater. 2018;7(9): Article e1701257. 29334196 10.1002/adhm.201701257

[34] Zhang H. Apatinib for molecular targeted therapy in tumor. Drug Des Devel Ther. 2015;9:6075–6081. 10.2147/DDDT.S97235 PMC4654530 26622168

[35] Zhu Z, Li S, Wu D, Ren H, Ni C, Wang C, Xiang N, Ni Z. High-throughput and label-free enrichment of malignant tumor cells and clusters from pleural and peritoneal effusions using inertial microfluidics. Lab Chip. 2022;22:2097–2106. 35441644 10.1039/d2lc00082b

[36] Yang C, Qin S. Apatinib targets both tumor and endothelial cells in hepatocellular carcinoma. Cancer Med. 2018;7(9):4570–4583. 30109780 10.1002/cam4.1664 PMC6144148

[37] Li G, Lin H, Tian R, Zhao P, Huang Y, Pang X, Zhao L, Cao B. VEGFR-2 inhibitor apatinib hinders endothelial cells progression triggered by irradiated gastric cancer cells-derived exosomes. J Cancer. 2018;9(21):4049–4057. 30410610 10.7150/jca.25370 PMC6218785

[38] Gorodecki J, Mortel R, Ladda RL, Ward SP, Geder L, Rapp F. Establishment and characterization of a new endometrial cancer cell line (SCRC-1). Am J Obstet Gynecol. 1979;135(5):671–679. 228555 10.1016/s0002-9378(16)32994-5

[39] Fadare O, Desouki MM, Gwin K, Hanley KZ, Jarboe EA, Liang SX, Quick CM, Zheng W, Parkash V, Hecht JL. Frequent expression of Napsin A in clear cell carcinoma of the endometrium: Potential diagnostic utility. Am J Surg Pathol. 2014;38(2):189–196. 24145649 10.1097/PAS.0000000000000085

[40] Hoang L, Han G, McConechy M, Lau S, Chow C, Gilks CB, Huntsman DG, Köbel M, Lee CH. Immunohistochemical characterization of prototypical endometrial clear cell carcinoma—Diagnostic utility of HNF-1β and oestrogen receptor. Histopathology. 2014;64:585–596. 24103020 10.1111/his.12286

[41] Igbinigie E, Guo F, Jiang S-W, Kelley C, Li J. Dkk1 involvement and its potential as a biomarker in pancreatic ductal adenocarcinoma. J Comput Biol. 2019;488:226–234. 10.1016/j.cca.2018.11.023 30452897

[42] Zhang P, Shao N, Qin L. Recent advances in microfluidic platforms for programming cell-based living materials. Adv Mater. 2021;33(46):2005944. 10.1002/adma.202005944 34270839

[43] You L, Pin-Rui S, Betjes M, Rad RG, Chou T-C, Beerens C, van Oosten E, Leufkens F, Gasecka P, Muraro M, et al. Linking the genotypes and phenotypes of cancer cells in heterogenous populations via real-time optical tagging and image analysis. Nat Biomed Eng. 2022;6:667–675. 35301448 10.1038/s41551-022-00853-x

[44] Silvestri VL, Henriet E, Linville RM, Wong AD, Searson PC, Ewald AJ. A tissue-engineered 3D microvessel model reveals the dynamics of mosaic vessel formation in breast cancer. Cancer Res. 2020;80:4288–4301. 32665356 10.1158/0008-5472.CAN-19-1564 PMC7541732

[45] Maishi N, Hida K. Tumor endothelial cells accelerate tumor metastasis. Cancer Sci. 2017;108:1921–1926. 28763139 10.1111/cas.13336 PMC5623747

[46] Ren Y, Wang T, Cheng X, Ke G, Huang Y, Yang H, Huang X, Tian W, Wang H. Efficacy and safety of apatinib in patients with recurrent uterine malignancy: A prospective, single-center, single-arm, phase 2 study. Ann Transl Med. 2023;11(2):106. 36819505 10.21037/atm-22-6463 PMC9929780

[47] Parlato S, Grisanti G, Sinibaldi G, Peruzzi G, Casciola CM, Gabriele L. Tumor-on-a-chip platforms to study cancer-immune system crosstalk in the era of immunotherapy. Lab Chip. 2021;21:234–253. 33315027 10.1039/d0lc00799d

[48] Anderson NM, Celeste Simon M. The tumor microenvironment. Curr Biol. 2020;30(16):921–925. 10.1016/j.cub.2020.06.081 PMC8194051 32810447

