# Supplementary material for: Engineered Endometrial Clear Cell Cancer-on-a-Chip Reveals Early Invasion-Metastasis Cascade of Cancer Cells
Source: Biomater Res. 2025 Apr 14;29:0177. doi: 10.34133/bmr.0177 (PMC11994883; doi:10.34133/bmr.0177)
Supplement: Supplementary 1 — Table S1 Figs. S1 to S9 [file bmr.0177.f1.docx]

**Engineered endometrial clear cell cancer on-a-chip reveals early invasion-metastasis cascade of cancer cells**

Chengpan Li^1#^, Jing Pan^2#^, Zhengdi Shi^1^, Xinyan Zeng^3^, Xiaoping Xia^4^, Xiaogang He^5^, Wei Wang^2^, Bensheng Qiu^1*^, Weiping Ding^2*^, Dabing Huang^2*^

^1^ Department of Electronic Engineering and Information Science, School of Information Science and Technology, University of Science and Technology of China, Hefei, Anhui 230027, China

^2^ Department of Oncology, The First Affiliated Hospital of USTC, Division of Life Sciences and Medicine, University of Science and Technology of China, Hefei, Anhui 230001, China

^3^ Department of Integrated Traditional Chinese and Western Medicine, Anhui Medical University, Hefei, Anhui 230032, China

^4^ Department of Obstetrics and Gynecology, Anhui Provincial Children’s Hospital, Children’s Hospital of Fudan University Anhui Hospital, Children’s Hospital of Anhui Medical University, Hefei, Anhui 230022, China

^5^ Department of Urology, The First Affiliated Hospital of USTC, Division of Life Sciences and Medicine, University of Science and Technology of China, Hefei, Anhui 230001, China

# These authors contributed equally.

*Corresponding authors:

Dabing Huang: hdabing@ustc.edu.cn; ORCID: 0000-0002-3040-3017

Weiping Ding: wpdings@ustc.edu.cn; ORCID: 0000-0002-3331-1011

Bensheng Qiu: [bqiu@ustc.edu.cn](mailto:bqiu@ustc.edu.cn); ORCID: [0000-0003-2987-7378](http://orcid.org/0000-0003-2987-7378)

**Table S1** Short tandem repeat (STR) results (there are no matches).


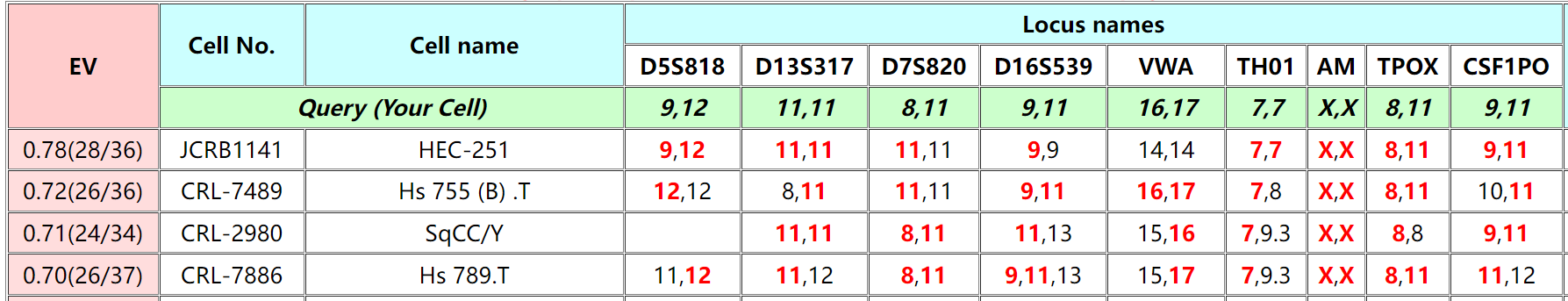


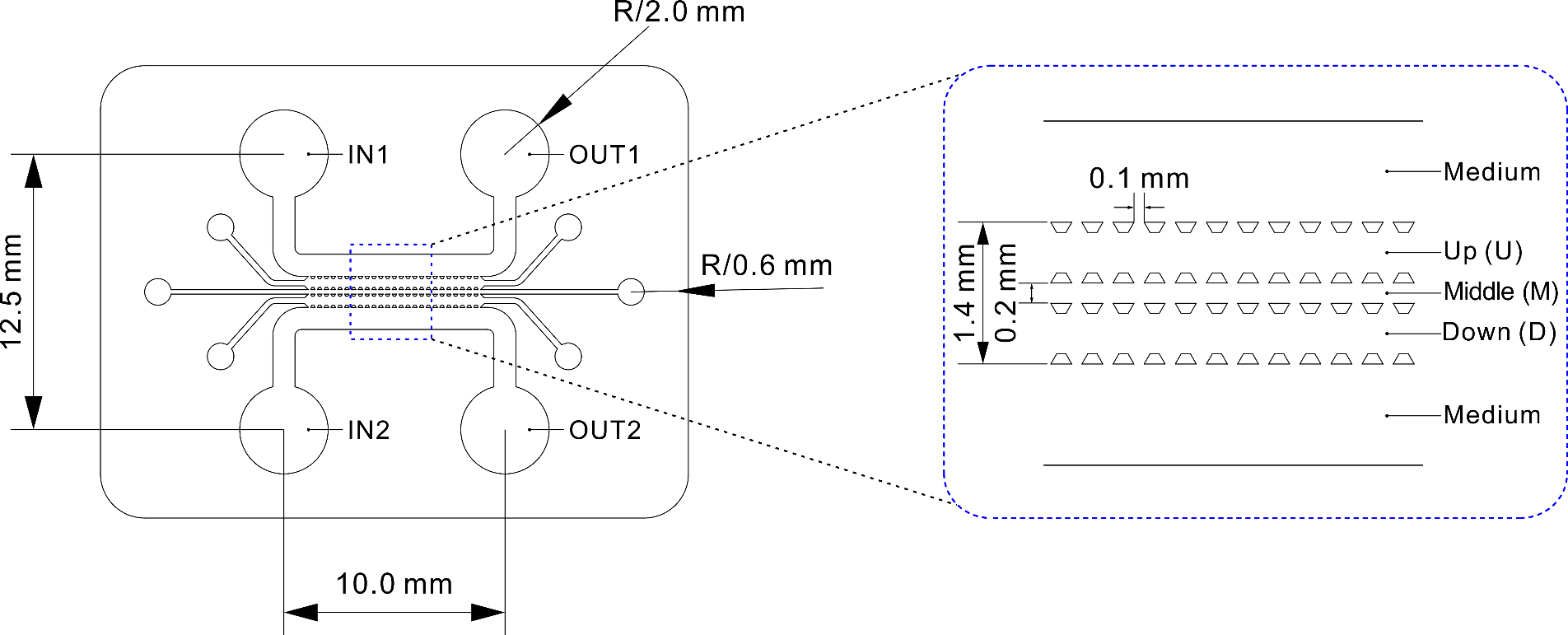


**Figure S1** Design parameters of the chip.


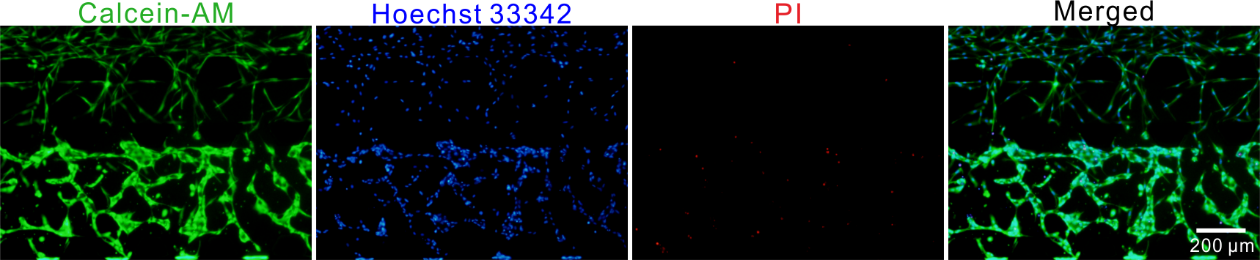


**Figure S2** On-chip cell viability. Living cells were stained with Calcein-AM dye (green), cell nuclei were labelled with Hoechst 33342 dye (blue), and dead cells were indicated with PI dye (red).


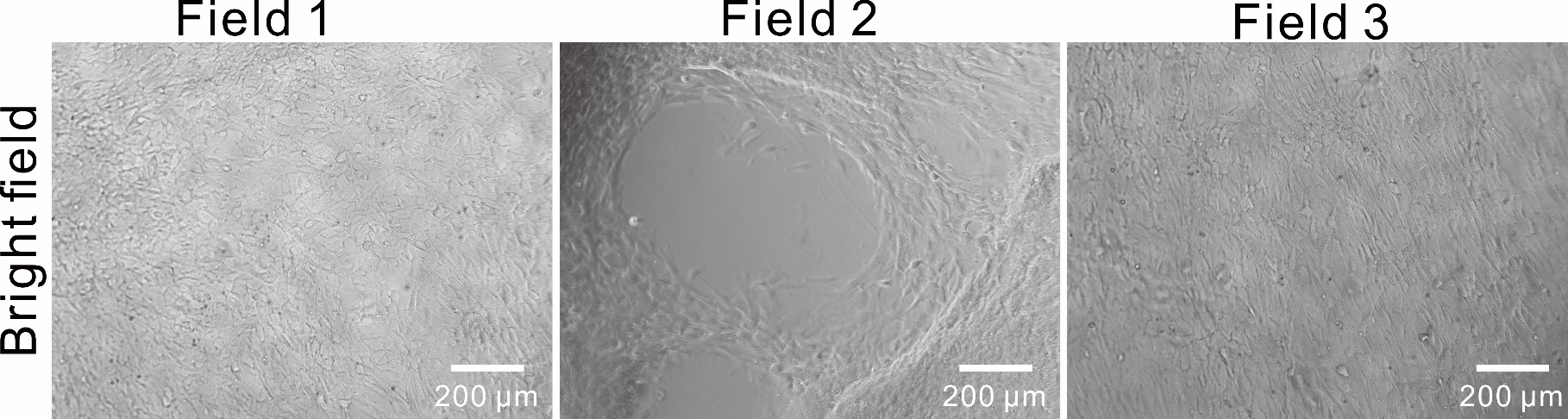


**Figure S3** Bright-field images of cells under static culture condition.


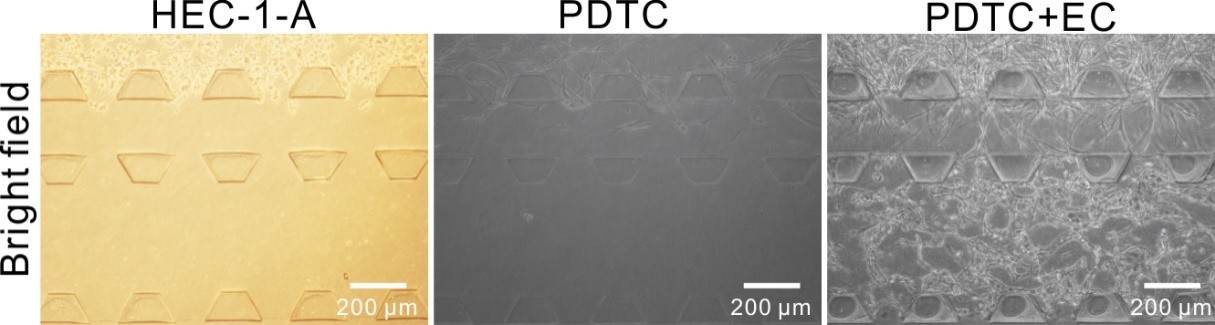


**Figure S4** Bright-field images of on-chip cells on day 6.


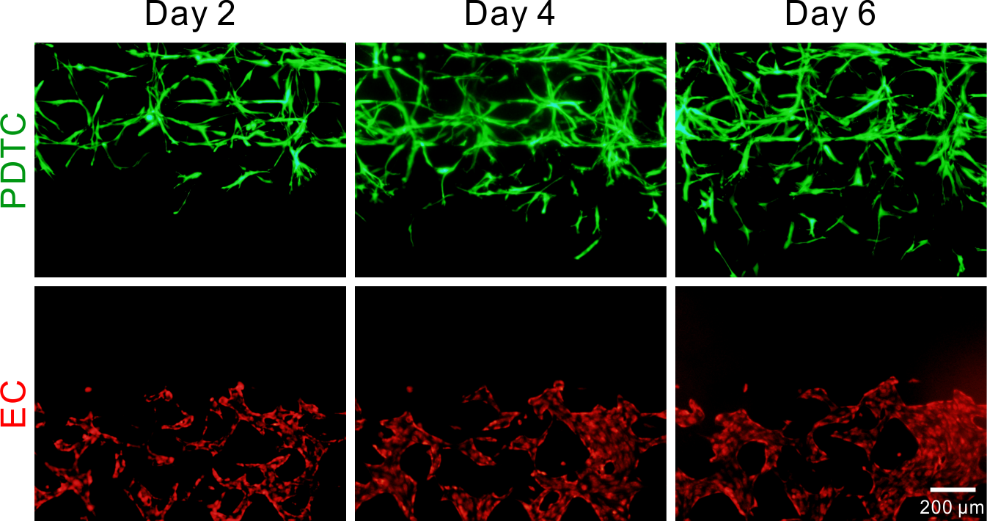


**Figure S5** Fluorescence images of on-chip PDTCs and ECs on days 2, 4 and 6.


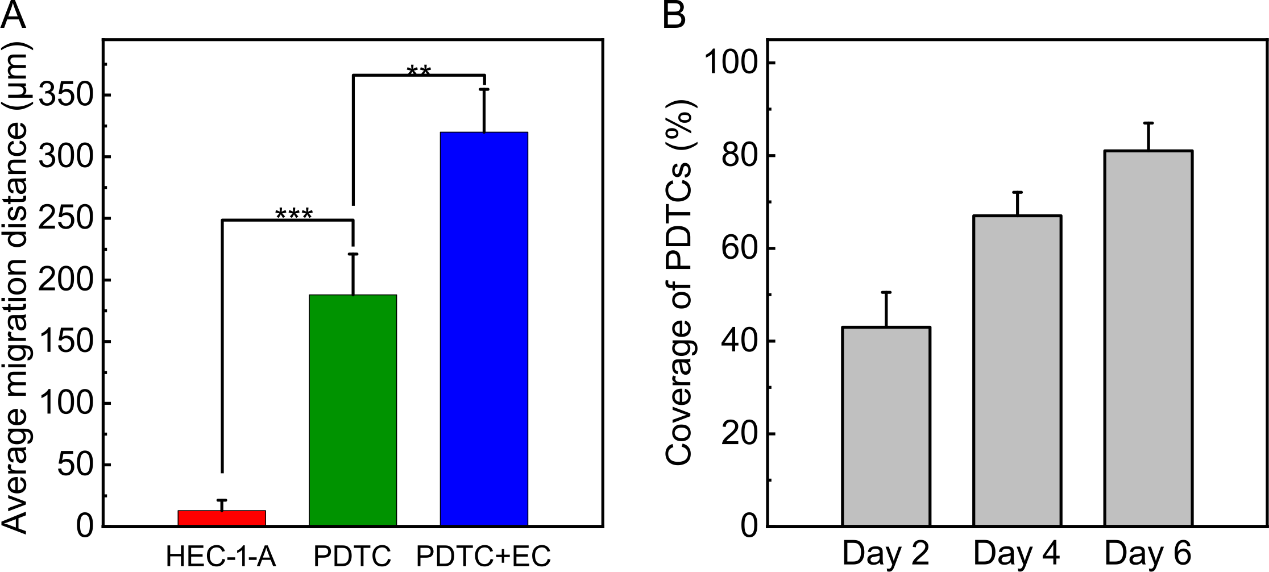


**Figure S6** (**A**)The average migration distance of cancer cells under different conditions and (**B**) the coverage of PDTCs on days 2, 4 and 6.


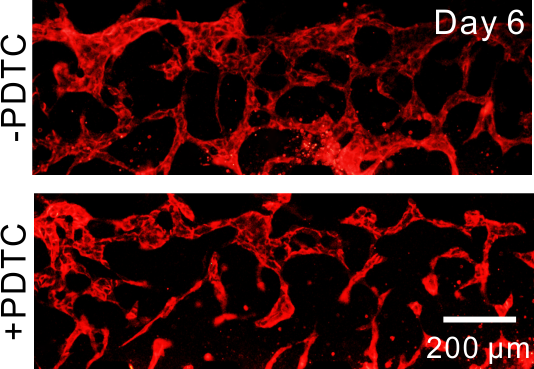


**Figure S7** Fluorescence images of microvessels when ECs were cultured with/without PDTCs. ECs were stained with CD31 antibody.


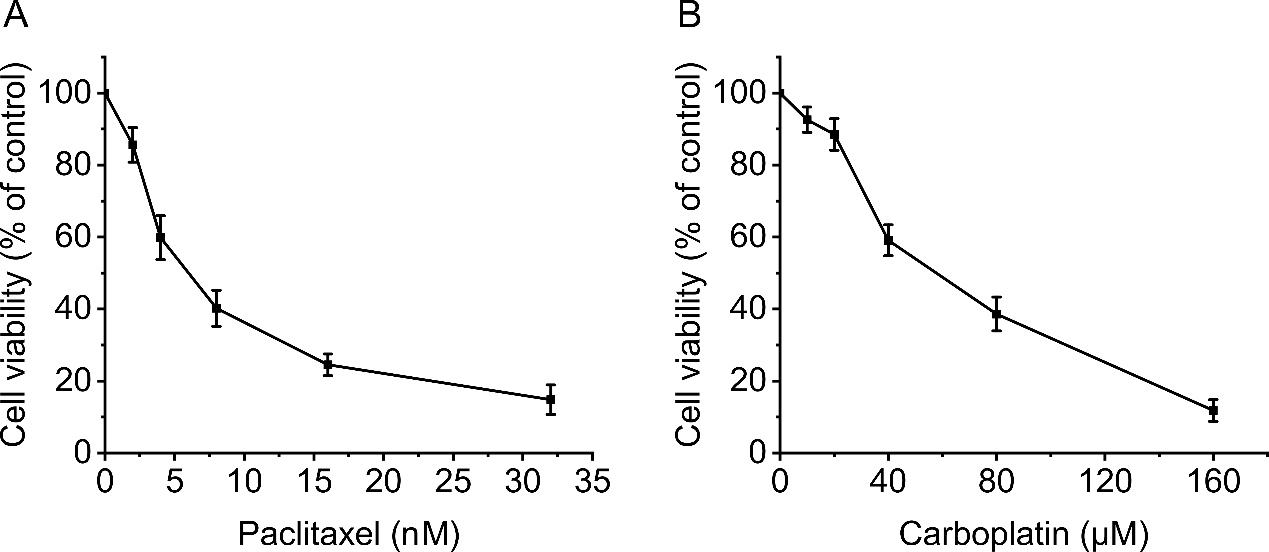


**Figure S8** The viability of cells treated with (**A**) paclitaxel and (**B**) carboplatin.


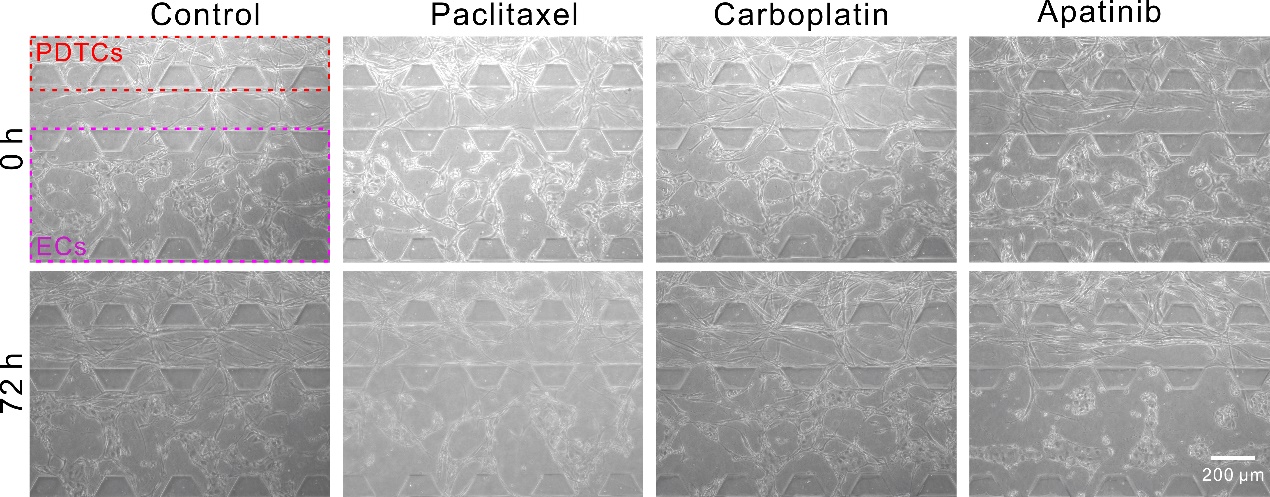


**Figure S9** Images of on-chip cells at different times in the control and drug-treated groups.
